# Quantitative analysis of m^6^A RNA modification by LC-MS

**DOI:** 10.1016/j.xpro.2021.100724

**Published:** 2021-08-06

**Authors:** Lavina Mathur, Sunhee Jung, Cholsoon Jang, Gina Lee

**Affiliations:** 1 Department of Microbiology and Molecular Genetics, Chao Family Comprehensive Cancer Center, University of California Irvine School of Medicine, Irvine, CA 92697-4025, USA; 2 Department of Biological Chemistry, Chao Family Comprehensive Cancer Center, University of California Irvine School of Medicine, Irvine, CA 92697-4025, USA

**Keywords:** cell biology, cell culture, metabolism, molecular biology, mass spectrometry, chemistry

## Abstract

*N*^6^-adenosine methylation (m^6^A) of messenger RNA (mRNA) plays key regulatory roles in gene expression. Accurate measurement of m^6^A levels is thus critical to understand its dynamic changes in various biological settings. Here, we provide a protocol to quantitate the levels of adenosine and m^6^A in cellular mRNAs. Using nuclease and phosphatase, we digest mRNA into nucleosides, which are subsequently quantified using liquid chromatography mass spectrometry.

For complete details on the use and execution of this protocol, please refer to Cho et al. (2021).

## Highlights

• Straightforward protocol for quantitation of m^6^A levels in cellular mRNA
• Purified mRNA is processed to nucleosides by nuclease and phosphatase
• Modified and unmodified nucleoside levels are measured by LC-MS
• The protocol can be applied to quantitate various chemical modifications in RNA and DNA

## Graphical Abstract

**Figure:**
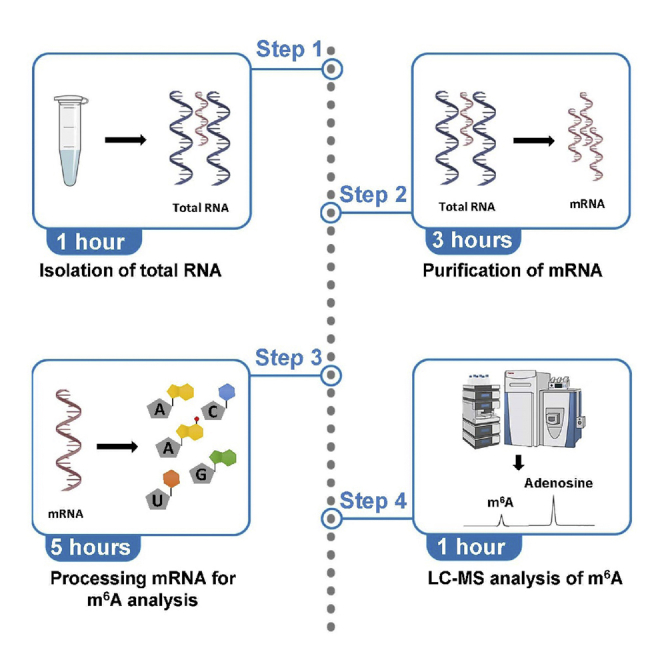

## Before you begin

This protocol requires preparation of several buffers and enzymes beforehand. To prevent degradation of RNA samples, it is necessary to follow general precautions for RNA experiments including preparation of RNase-free plastic wares and wiping working surfaces with RNase inactivating agents. To avoid contamination of buffers with RNase, we recommend that the users purchase RNase-free buffers (list provided in the key resources table). Using these raw materials, prepare working solutions and enzyme mixtures as described in the Materials and Equipment Section. The quality of mass spectrometry reagents (e.g., organic solvents, water) is also critical to reduce contamination of RNA and nucleotides from external sources.

### HEK293E cell culture

**Timing: 3 days**

The protocol was used to measure m^6^A levels in HEK293E cell line but can be adapted for any cells and tissue samples. Prepare enough number of cells and tissues to isolate >50 μg total RNA.

1. Seed around 350,000 cells in a 60 mm cell plate.
2. After two days, harvest the cells for total RNA isolation.

## Key resources table

**Table:** 

REAGENT or RESOURCE	SOURCE	IDENTIFIER
**Chemicals, buffers and enzymes**		

*N*^6^-methyladenosine (m^6^A)	Selleckchem	Cat#S3190
Adenosine	Sigma-Aldrich	Cat#A9251
10× Phosphate Buffered Saline (PBS)	Corning	Cat#46-013-CM
EDTA 0.5 M (pH8.0)	Promega	Cat#V4231
Glycerol	Sigma-Aldrich	Cat#G5516-100ML
LiCl 8 M	Sigma-Aldrich	Cat#L7026-100ML
MgCl_2_ 25 mM	New England Biolabs (NEB)	Cat#B9021S
ZnCl_2_ 0.1M	Sigma-Aldrich	Cat#39059-1ML-F
NaCl 5M	Quality Biological Inc	Cat#351-036-721EA
Ammonium Bicarbonate	Sigma-Aldrich	Cat#09830
Nuclease-free water	HyClone	Cat#SH30538.FS
200 Proof Ethanol	Sigma-Aldrich	Cat#459836
RNase decontamination solution	Genesee Scientific	Cat#10-456
PCR grade water	IBI Scientific	Cat#IB42301
Sodium acetate 3 M (pH5.2)	Corning	Cat#46-033-CI
Tris Hydrochloride (Tris-HCl) 1 M (pH7.5)	Fisher Scientific	Cat#BP1757-500
Beta-Mercaptoethanol	Sigma-Aldrich	Cat#M3148-25ML
Hydrochloric Acid (HCl) 6 M	Fisher Scientific	Cat# S25857
Nuclease P1 from *Penicillium citrinum*	Sigma-Aldrich	Cat#N8630-1VL
Alkaline phosphatase from Escherichia coli	Sigma-Aldrich	Cat#P5931-100UN
Oligo(dT)_25_ magnetic beads	New England Biolabs	Cat#S1419S
Water (HPLC Grade)	Fisher Scientific	Cat#AA22934M6
Acetonitrile (HPLC Grade)	Fisher Scientific	Cat#6000247
Ammonium Acetate	Spectrum Chemical	Cat#A2149-1KG
Ammonium Hydroxide	Spectrum Chemical	Cat#A1195
23G Syringe	Fisher Scientific	Cat#309571
1.5 mL low-bind tube	Corning	Cat#3207
Cell scraper	Corning	Cat#3008
0.2 μm PES filter	Whatman Puradisc	Cat#6780-2502
1 mL Syringe	Becton Dickinson	Cat#329654
Snap Cap, pre-slit	Fisher Scientific	Cat#14-823-480
Stepvial System™ Crimp/Snap Vial	Fisher Scientific	Cat#05-704-225
Xbridge BEH amide column (150 × 2.1 mm, 3 μm particle size)	Waters	Cat#186006724
DMEM	Gibco	Cat#11965118
Fetal Bovine Serum (FBS)	Sigma-Aldrich	Cat#F0926-500ML

**Critical commercial assays**		

PureLink RNA Mini Kit	Ambion	Cat#12183018A
RNA Clean & Concentrator kit	Zymo Research	Cat#R1016

**Experimental models: Cell line**		

Human: HEK293E	ATCC	Cat#293c18; RRID: CVCL_6974

**Software**		

Xcalibur	Thermo Scientific	n/a
ProteoWizard	ProteoWizard	n/a
MAVEN	Elucidata	n/a

**Other**		

Refrigerated centrifuge	Eppendorf	Cat#2231000655
Chemical Hood	Labconco	Cat#3746704
Magnetic Rack	Bio-Rad	Cat#1614916
Mini centrifuge	Benchmark Scientific	Cat#C1008-R
NanoDrop 2000c	Thermo Scientific	Cat#ND2000C
Thermomixer C	Eppendorf	Cat#5382000023
Dry heat block	Benchmark Scientific	Cat#BSH6000
Vortex	Benchmark Scientific	Cat#BV1003
Thermo Q Exactive Plus Hybrid Quadrupole-Orbitrap Mass Spectrometer	Thermo Scientific	n/a
Vanquish UHPLC System	Thermo Scientific	n/a

## Materials and equipment

Below is the list of reagents that need to be prepared before experiments. Use raw materials in the key resources table or other available reagents with similar grade (i.e., RNase-free materials). Individual procedures take 10–30 min. The buffers and reconstituted enzymes are good for use for 3–6 months.

### PureLink RNA Mini Kit

The kit provides PureLink Lysis Buffer, Wash Buffer I, Wash Buffer II, and Spin cartridges for total RNA isolation. Reconstitute the Lysis Buffer with beta-mercaptoethanol and Wash Buffer II with ethanol according to the manufacturer’s protocol.

### Zymo RNA Clean & Concentrator Kit

The kit provides Zymo RNA Binding Buffer, RNA Prep Buffer, RNA Wash Buffer, and Spin cartridges for removal of salts from RNA samples. Reconstitute the RNA Wash Buffer with ethanol according to the manufacturer’s protocol.

### Oligo(dT) Binding Buffer

After mixing below components, store the buffer at 4°C.

**Table:** 

Reagent	Stock concentration	Final concentration	Amount
Tris-HCl (pH7.5)	1 M	20 mM	1 mL
LiCl	8 M	1 M	6.25 mL
EDTA	500 mM	2 mM	200 μL
Nuclease-free water	n/a	n/a	42.55 mL
**Total**	**n/a**	**n/a**	**50 mL**

### Oligo(dT) wash buffer

After mixing below components, store the buffer at 4°C.

**Table:** 

Reagent	Stock concentration	Final concentration	Amount
Tris-HCl (pH7.5)	1 M	10 mM	500 μL
LiCl	8 M	150 mM	937.5 μL
500 mM EDTA	500 mM	1 mM	100 μL
Nuclease-free water	n/a	n/a	48.5 mL
**Total**	**n/a**	**n/a**	**50 mL**

### Oligo(dT) Elution Buffer

Dilute 100 μL of 1M Tris-HCl (pH7.5) with 9.9 mL nuclease-free water to make 10 mM Tris-HCl. Store at 4°C.

### Reconstitution of nuclease P1

Prepare 2 unit/μL nuclease P1 stock by dissolving nuclease P1 powder in nuclease P1 reconstitution buffer. Aliquot into 10 μL and store at −20°C.

### Nuclease P1 reconstitution buffer

After mixing below components, store the buffer at 4°C.

**Table:** 

Reagent	Stock concentration	Final concentration	Amount
NaOAc (pH5.3)	3 M	50 mM	60 μL
ZnCl2	100 mM	1 mM	100 μL
Glycerol	100%	25%	2.5 mL
Nuclease-free water	n/a	n/a	7.34 mL
**Total**	**n/a**	**n/a**	**10 mL**

### Reconstitution of alkaline phosphatase

Prepare 2 unit/μL alkaline phosphatase stock by dissolving alkaline phosphatase powder in alkaline phosphatase reconstitution buffer. Aliquot into 10 μL and store at −20°C.

### Alkaline phosphatase reconstitution buffer

After mixing below components, store the buffer at 4°C.

**Table:** 

Reagent	Stock concentration	Final concentration	Amount
Tris-HCl (pH7.5)	1 M	5 mM	50 μL
MgCl_2_	25 mM	0.5 mM	200 μL
Glycerol	100%	25%	2.5 mL
Nuclease-free water	n/a	n/a	7.35 mL
**Total**	**n/a**	**n/a**	**10 mL**

### 2 M ammonium bicarbonate

Dissolve 158 mg ammonium bicarbonate in 1 mL PCR grade water. Filter through 0.2 μM PES filter using 1 mL syringe. Prepare fresh ammonium bicarbonate solution on the day of experiment.

### 1.2 M HCl

Dilute 100 μL 6 M HCl in 500 μL PCR grade water. Store at 4°C.

### Preparation of m^6^A and adenosine standards

Dissolve 1 mg of m^6^A or adenosine powder in 1 mL of 75% acetonitrile (acetonitrile: water, 75:25, v/v). Dilute each standard as 1 mg/L. Mix m^6^A and adenosine standard solutions with the same volume (1:1) to make a standard solution containing 500 μg/L of both m^6^A and adenosine. Aliquot this standard solution mix into 100 μL and store at −80°C. To make standard calibration curves, make serial dilution of standards at 0.5, 1, 2, 5, 10, 20 and 50 μg/L. Then, obtain *y* = *ax* equation (*a* is constant) with ion counts (*y*) and standard concentrations (*x*) using linear regression.

### LC-MS setting

Thermo Q Exactive™ Plus Hybrid Quadrupole-Orbitrap™ Mass Spectrometer coupled with Vanquish UHPLC system was used. LC-MS system was controlled by Xcalibur software (Thermo). Metabolite separation was conducted by Xbridge BEH amide column (150 × 2.1 mm, 3 μm particle size). LC gradient was generated using LC solvents A and B (Table 1). Autosampler temperature was set at 4°C and the column temperature was set at 25°C. MS analysis was performed with a full-scan mode for measurement of samples (m/z range 250–300, positive ion mode). MS^2^ fragmentation was used to confirm m^6^A and adenosine (Table 2).

**Table 1:** LC gradient method

min	Flow (mL/min)	LC solvent A (%)	LC solvent B (%)
0	0.35	25	75
3	0.35	25	75
4	0.35	50	50
5	0.35	90	10
7	0.35	90	10
7.5	0.35	25	75
11	0.35	25	75

**Table 2:** MS parameters

Parameter	Value
Sheath gas flow rate	40 psi
Aux gas flow rate	10 psi
Sweep gas flow rate	2 psi
Spray voltage	2.7 kV
Capillary temperature	300°C
Collision energy	40 eV
Peak width	6 s
S-lens RF level	50
AGC target	3E+06
Maximum injection time	500 msec

### LC solvent A

After mixing below components, store the solvent at 25°C.

**Table:** 

Reagent	Stock concentration	Final concentration	Amount
Ammonium acetate dissolved in water	1 M	20 mM	10 mL
Ammonium hydroxide dissolved in water	1 M	20 mM	10 mL
Acetonitrile	100%	5%	25 mL
Water	n/a	n/a	455 mL
**Total**	**n/a**	**n/a**	**500 mL**

### LC solvent B

100% Acetonitrile

## Step-by-step method details

### Isolation of total RNA

**Timing: 1 h**

In this step, total RNA is isolated from the cells using PureLink RNA Mini Kit. Prepare the buffers in the kit according to the manufacturer’s protocol before starting the experiment.

1. Sample harvest and homogenization
  a. Remove medium from cells and rinse with 1× PBS (e.g., 5 mL for 60 mm plates).
  b. Add 350 μL of PureLink Lysis buffer to the plate. Scrape the cell lysate thoroughly using a cell scraper.
  c. Transfer the viscous liquid into a new 1.5 mL tube.
    **Pause point:** Samples can be frozen at −80°C.
  d. Homogenize the sample with a 23G syringe needle. Repeat the suction-release step 5–10 times.
    ***Note:*** Try not to generate too many bubbles during homogenization (samples can overflow the tubes).

2. Purification of total RNA
  a. Add 350 μL of 70% ethanol to sample (Sample: 70% ethanol = 1:1) and vortex.
  b. Transfer 700 μL of the sample into the PureLink Spin cartridge and centrifuge for 15 s at 12,000 × *g* at 25°C. Discard the flow through.
  c. Add 700 μL of PureLink Wash Buffer I and centrifuge for 15 s at 12,000 × *g* at 25°C. Discard the flow through.
  d. Add 500 μL of PureLink Wash Buffer II and centrifuge for 15 s at 12,000 × *g* at 25°C. Discard the flow through. Repeat the step twice.
  e. Centrifuge the column for 2 min at 12,000 × *g* to ensure complete removal of the wash buffer.
  f. Transfer the column to a new 1.5 mL tube.
    ***Note:*** Leave the column on the tube for 5 min to **evaporate** any residual ethanol from the wash buffer.
  g. Add 50 μL nuclease-free water directly to the column matrix and incubate for 5 min.
  h. Centrifuge for 2 min at 12,000 × *g* at 25°C. The flow through contains total RNA.
    **Pause point:** Samples can be frozen at −80°C.

3. Measure RNA concentration using Nanodrop with absorbance at 260 nm.

**Note:** 80–90% confluent HEK293E cells from a 60 mm plate result in >75 μg total RNA.

### Purification of mRNA using Oligo(dT) beads

**Timing: 3 h**

In this step, polyadenylated [poly(A)] mRNA is isolated from total RNA using oligo(dT) beads. Except heat block and ice incubation steps, all procedures are performed at 25°C. When not in the reaction (i.e., while preparing beads or kits), RNA samples should be kept on ice. Before starting the experiment, bring the oligo(dT) Binding and oligo(dT) Wash buffers to 25°C, and reconstitute the buffers in the Zymo RNA Clean & Concentrator kit.

4. Preparation of RNA
  a. Spin down the samples.
  b. Adjust the sample amount to contain 50 μg of total RNA in 100 μL nuclease-free water.
  c. Heat the samples in dry heat block at 65°C for 2 min to disrupt RNA secondary structures.
  d. Place the sample on ice immediately.

***Note:*** Rapid cool down of the heated RNA samples is the key to minimize secondary structures for efficient binding of RNAs with Oligo(dT) beads.

5. Preparation of Oligo(dT) beads
  a. Resuspend Oligo(dT) beads (vortex >30 s or tilt for 5 min).
  b. Transfer beads to a new 1.5 mL tube. Use 250 μg beads for 50 μg total RNA.
    ***Note:*** For example, the concentration of NEB #S1419S bead suspension is 5 mg/mL. Use 50 μL of NEB #S1419S bead suspension to get 250 μg beads.
  c. Add 0.5 mL Oligo(dT) Binding Buffer to the beads and rinse by pipetting.
  d. Place the tube on the magnetic rack until the solution is clear (i.e., incubate for 1 min on the magnetic rack).
  e. Carefully remove the supernatant using a pipette.
    ***Note:*** Do not use aspirator to prevent loss of the beads.
  f. Add 200 μL Oligo(dT) Binding Buffer to the beads and mix well.

6. Isolation of mRNA
  a. Add 100 μL RNA solution (prepared in step 4) to 200 μL Oligo(dT) bead suspension (i.e., RNA solution: Bead suspension = 1:2). Mix thoroughly by pipetting.
  b. Incubate the samples in Thermomixer for 5 min with agitation at 800 rpm at 25°C.
  c. Place the tube on the magnetic rack until the solution is clear, and then remove the supernatant.
  d. Wash the mRNA-bead complex with 200 μL Oligo(dT) Wash Buffer by pipetting.
    ***Note:*** When handling multiple samples, stagger steps 6a–6d to decrease differences in bead incubation times among the samples.
  e. Incubate on the magnetic rack and remove the supernatant.
  f. Repeat steps 6d and 6e.
  g. To discard the wash buffer completely, centrifuge at 200 × *g* for 10 s at 25°C.
    ***Note:*** Do not centrifugate the beads at speeds higher than 200 × *g*. Place the tube in a metal rack and remove the residual wash buffer.

7. Elution
  a. Add 50 μL of Oligo(dT) Elution Buffer to the beads. Mix well by pipetting.
  b. To elute mRNA from the beads, heat the samples at 75°C for 2 min.
  c. Immediately place the tube on the magnetic rack and incubate until the solution is clear.
  d. Transfer the supernatant (i.e., eluted mRNAs) to a new 1.5 mL tube.

**Pause point:** Samples can be frozen at −80°C.

8. Repeat one more round of Oligo(dT) purification (i.e., steps 4–7) to achieve higher mRNA purity.

**Pause point:** Samples can be frozen at −80°C.

9. Conduct RNA clean-up using Zymo RNA Clean & Concentrator kit to remove residual salts from mRNA samples for the m^6^A processing step.
  a. Add 100 μL Zymo RNA Binding Buffer to 50 μL mRNA sample and mix (RNA Binding Buffer: mRNA sample = 2:1).
    ***Note:*** To decrease variations in the isolated mRNA amount among the samples, use same amount of total RNA as a starting material (e.g., Adjust total RNA amount as 50 μg across all samples).
  b. Add 150 μL of 100% ethanol and mix (mRNA-RNA Binding Buffer: 100% ethanol = 1:1)
  c. Transfer the sample to the Zymo Spin cartridge.
  d. Centrifuge at 12,000 × *g* for 30 s at 25°C. Discard the flow through.
  e. Add 400 μL Zymo RNA Prep Buffer to the column and centrifuge at 12,000 × *g* for 30 s at 25°C. Discard the flow through.
  f. Add 700 μL Zymo RNA Wash Buffer to the column and centrifuge at 12,000 × *g* for 30 s at 25°C. Discard the flow through.
  g. Add 400 μL Zymo RNA Wash Buffer to the column and centrifuge for 2 min to completely remove the wash buffer.
  h. Transfer the column carefully into a new 1.5 mL tube.
    ***Note:*** Leave the column on the tube for 5 min to evaporate any residual ethanol from the wash buffer.
  i. Add 15 μL nuclease-free water directly to the column matrix and incubate for 5 min.
  j. Centrifuge at 16,000 × *g* for 30 s at 25°C. The flow through contains purified mRNA.
    **Pause point:** Samples can be frozen at −80°C.

10. Measure mRNA concentration using Nanodrop with absorbance at 260 nm. 50 μg of total RNA results in >300 ng mRNA.

### Processing of mRNA samples for m^6^A analysis

**Timing: 5 h**

In this step, nuclease P1 hydrolyzes phosphodiester bonds in mRNA to generate nucleoside 5′-monophosphates (nucleotides). Nucleotides are further processed with alkaline phosphatase to remove phosphate groups for LC-MS analysis. Efficient enzymatic processing is critical for the LC-MS to accurately detect each nucleoside signal. Use 100–200 ng of mRNAs for sample processing. Include negative control (water-only sample) to estimate and subtract background signals.

11. Nuclease P1 digestion
  a. In each sample tube, add the components listed below. Make a master mix and distribute to the samples to reduce pipetting error.

**Table:** 

Reagent	Stock concentration	Final concentration	Amount
mRNA	n/a	200 ng	20 μL
Nuclease P1	2 unit/μL	1 unit	0.5 μL
NaCl	5 M	25 mM	0.4 μL
ZnCl_2_	0.1 M	2.5 mM	2 μL
PCR grade water	n/a	n/a	17.1 μL
**Total**	**n/a**	**n/a**	**40 μL**
  b. Vortex briefly and spin down the samples.
  c. Incubate the samples in Thermomixer for 2 h at 37°C with agitation at 800 rpm for 30 s every 5 min.

12. Phosphatase treatment
  a. Add 2 μL of 2 M ammonium bicarbonate solution.
  b. Vortex briefly and spin down the samples.
  c. Add 1 unit of alkaline phosphatase.
  d. Vortex briefly and spin down the samples.
  e. Incubate the samples in Thermomixer for 2 h at 37°C with agitation at 800 rpm for 30 s every 5 min.
13. Re-neutralization of the solution
  a. To neutralize the reaction, add 1 μL of 1.2 M HCl. Vortex briefly to mix the samples.
  b. Centrifuge the samples for 30 min at 16,000 × *g* at 4°C to precipitate any insoluble parts.
  c. Transfer 20 μL supernatant to a new 1.5 mL tube.

**Pause point:** Samples can be frozen at −80°C.

### LC-MS analysis of m^6^A

**Timing: 1 h**

14. Preparation of LC-MS samples
  a. Mix 20 μL of the purified nucleoside samples with 40 μL of acetonitrile.
    ***Note:*** This is to have a similar proportion of organic solvent in the LC-MS sample with the starting LC mobile phase (75% acetonitrile).
  b. Centrifuge the samples at 16,000 × *g* for 10 min at 4°C to precipitate any insoluble parts.
  c. Carefully transfer 40 μL of supernatant to a new LC-MS vial.
    ***Note:*** Do not touch the pellet.

15. Inject 3 μL of samples to the LC-MS system with the setting parameters described above.

***Note:*** The 3 μL sample now contains ∼4.5 ng mRNA if the m^6^A processing was performed with 200 ng mRNA as a starting material.

16. Run m^6^A and adenosine standards in the same LC-MS setting with the samples.

## Expected outcomes

**Figure 1:**
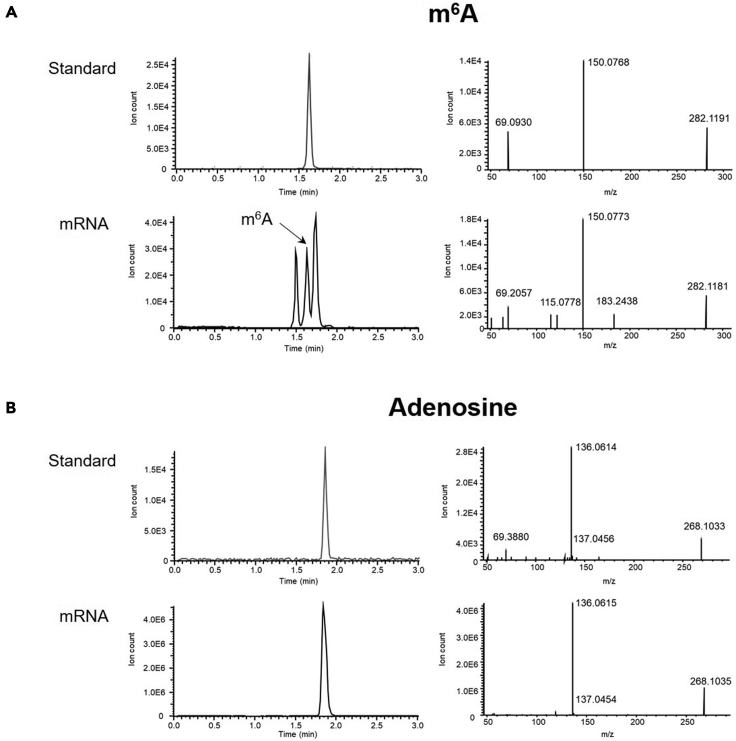
LC-MS chromatograms of m^6^A and adenosine Representative LC-MS (left) and MS/MS (right) chromatogram of m^6^A (A) and adenosine (B) derived from standards and purified mRNA samples (see also Table 3).

Under suggested conditions, m^6^A and adenosine are eluted at 1.65 min and 1.86 min, respectively (Figure 1 and Table 3).

**Table 3:** Summary of LC-MS results (see also Figure 1)

Metabolite	Formula	m/z	Ion species	Retention time (min)
*N*^6^-Methyladenosine (m^6^A)	C11H15N5O4	282.1197	[M+H]+	1.65
Adenosine	C10H13N5O4	268.1041	[M+H]+	1.86

## Quantification and statistical analysis

1. Convert LC-MS raw data files to mzXML using Proteowizard software.

***Note:*** MAVEN software (https://resources.elucidata.io/elmaven) or other software can be used to use mzXML file for peak visualization and quantitation.

**Figure 2:**
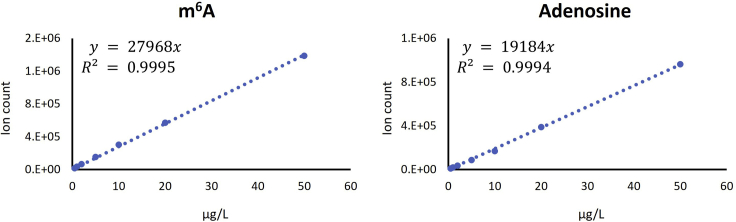
Standard calibration curves of m^6^A and adenosine.

2. Export ion counts of m^6^A and adenosine for each sample.
3. Calculate the concentration of m^6^A and adenosine using standard calibration curves (Figure 2).

## Limitations

This protocol details quantitative measurement of m^6^A modification in mRNAs using LC-MS. While this protocol is straightforward and easy to follow, it has some limitations.

First, to measure m^6^A modification of mRNAs, we purified mRNA from total RNA. However, contamination of abundant RNA species such as ribosomal RNA (rRNA) can occur. To measure m^6^A levels specifically from the mRNA m^6^A modification sequence (GA∗C; A∗ is methylated adenosine), the users can adopt RNase T1-based assays such as 2D thin-layer chromatography (TLC) (Bodi and Fray, 2017). In the TLC assay, mRNAs are processed with RNase T1 (specifically cleaves after G) followed by ^32^P labeling of nucleotides, which enables specific labeling of m^6^A from mRNAs.

Second, this protocol quantitates m^6^A levels from a total pool of mRNAs and cannot distinguish differential m^6^A modification levels in individual genes. This requires site-specific m^6^A detection using qPCR or TLC. Transcriptome-wide m^6^A sequencing methods have also been developed by several groups (reviewed in Zaccara et al., 2019).

Finally, while this protocol provides an optimized LC-MS condition for efficient measurement of m^6^A and adenosine in mRNA, users can adjust RNA purification and mass spectrometry methods to quantitate other modifications in various RNA species and DNA (Su et al., 2014; Thüring et al., 2017; Wei et al., 2018; Wein et al., 2020). Comprehensive analysis of nucleotide chemical modifications using LC-MS technology will provide valuable tools and resources in the field of transcriptomics, genomics, and metabolomics.

## Troubleshooting

### Problem 1

Low yield of mRNA (Related step: purification of mRNA using Oligo(dT) beads).

### Potential solution

Since mRNA is only 1–5% of total RNA, preparation of enough amount of total RNA is key to get enough amount of mRNA (e.g., 30–100 μg total RNA as a starting material). Also, use nucleic acid low-bind tubes and low retention pipette tips to minimize loss of mRNAs during purification. We recommend calculating mRNA purification yield using the amount of starting material (total RNA, step 3) and final mRNA product (step 10; consider that the yield of Zymo RNA Clean & Concentrator Kit is 70–80%). To decrease differences in mRNA yield among the samples, stagger the 5 min Oligo(dT) bead-RNA incubation step when handling several samples. Randomization of the sample order during the reaction also helps to decrease the incubation time differences caused by the sample order (i.e., randomization of sample order prevents Sample #1, 2, 3 being incubated longer with the beads than Sample #22, 23, 24).

### Problem 2

Contamination of other RNA species (Related step: purification of mRNA using Oligo(dT) beads).

### Potential solution

Contamination of abundance RNA species such as rRNA can interfere with accurate measurement of m^6^A and adenosine levels from mRNAs. To increase the purity of mRNA, we recommend conducting the oligo(dT) bead isolation twice. For the same sample, beads can be reused after washing the beads with oligo(dT) Wash Buffer. To evaluate rRNA contamination, conduct qPCR with primers that are specific to rRNAs. rRNAs can be further removed by RiboMinus Eukaryote Kit (Invitrogen Cat#A15020).

### Problem 3

Background nucleoside signal (Related step: processing of mRNA samples for m6A analysis).

### Potential solution

Due to the nucleic acids contaminated from the environment and reagents, background m^6^A and adenosine signals can be detected. To prevent this, we recommend using PCR grade (i.e., nucleic acid-free) water during the m^6^A processing step and subtracting the background signals detected in the water-only sample. Also, conduct m^6^A processing step in a clean chemical fume hood.

### Problem 4

Confirmation of m^6^A peak (Related steps: LC-MS analysis of m6A and expected outcomes).

### Potential solution

Some samples may show m^6^A isomers as shown in the Figure 1A (left panel). To avoid mis-annotation of peaks, we recommend running m^6^A standards in parallel with the samples to obtain accurate retention time. MS/MS profile can be used to confirm correct m^6^A peak (Figure 1A, right panel).

### Problem 5

Alternative reagents and equipment (Related step: key resources table).

### Potential solution

In the key resources table, we provided catalog numbers of the reagents and equipment that this protocol used, which may not be available in other circumstances. Users can use any reagents with equivalent grade (e.g., RNase-free reagents for RNA isolation and processing steps; HPLC-grade reagents for LC-MS). Regarding the equipment, (1) Oligo(dT)-RNA sample can be mixed using orbital shakers or rotators instead of Thermomixer; (2) Shaking nuclease P1 and alkaline phosphatase reactions in Thermomixer is optional (i.e., reaction can be performed in a regular heat block without agitation); (3) for mass spectrometry analysis of nucleoside samples, any type of high-sensitivity tandem mass spectrometers such as triple quadrupole, quadrupole-time of flight, and quadrupole-orbitrap can be used.

## Resource availability

### Lead contact

Further information and requests for resources and reagents should be directed to and will be fulfilled by the lead contact, Gina Lee (ginalee@uci.edu).

### Materials availability

This study did not generate new unique materials, reagents, or cell lines.

## Data Availability

The published article includes all datasets generated and analyzed during this study.
